# Host habitat shapes the gut microbiomes of insular reptilian hosts in the Philippines

**DOI:** 10.1093/ismeco/ycaf141

**Published:** 2025-09-04

**Authors:** Sierra N Smith, Jason B Fernandez, Cameron D Siler

**Affiliations:** Sam Noble Oklahoma Museum of Natural History, 2401 Chautauqua Ave., Norman, OK 73072, United States; School of Biological Sciences, University of Oklahoma, 730 Van Vleet Oval, Room 314, Norman, OK 73019, United States; Department of Biology, University of Texas at Arlington, 501 S. Nedderman Dr. Box 19498, Life Science Bldg. Rm. 337, Arlington, TX 76019-0498, United States; Zone 1, Bogñabong, Tabaco City, Albay, Philippines; Sam Noble Oklahoma Museum of Natural History, 2401 Chautauqua Ave., Norman, OK 73072, United States; School of Biological Sciences, University of Oklahoma, 730 Van Vleet Oval, Room 314, Norman, OK 73019, United States

**Keywords:** ecology, evolutionary biology, islands, microbiomes, reptiles

## Abstract

Islands have long served as ideal, replicative “natural laboratories” to help identify the mechanisms that shape the diversity and distribution of plant and animal communities, and a burgeoning body of literature has utilized island-like systems to better understand the processes that shape microbial community diversity. Despite this expanded application, few studies have explored patterns of microbial diversity spanning true islands, especially among communities of microorganisms that colonize vertebrate hosts (i.e. microbiomes). Here, we use 16S ribosomal ribonucleic acid microbial inventories to elucidate the roles that host evolutionary history, host habitat, host microhabitat, and geographic location play in the assemblage of gut microbiomes among reptilian hosts spanning multiple islands in the Philippines. Host habitat and microhabitat explained most of the variation in gut microbiome diversity observed among our focal hosts. Although we identified some significant differences in microbiome diversity across two of the host suborders (Lacertilia and Serpentes) and some host families, we did not find evidence of phylogenetic signal. We also conducted analyses of microbiome diversity across various geographic scales, and found that hosts inhabiting the same island, but different localities, did not possess significantly different gut microbiomes. However, the gut microbial diversity of hosts inhabiting distinct islands were significantly different across numerous measures of microbiome diversity. Results from this robust, comparative study contribute to our growing knowledge of the host-associated and geographic mechanisms that shape the vertebrate gut microbiome and represents one of the first studies to characterize variation in gut microbial communities among vertebrate hosts inhabiting multiple Philippine islands.

## Introduction

**Figure 1:**
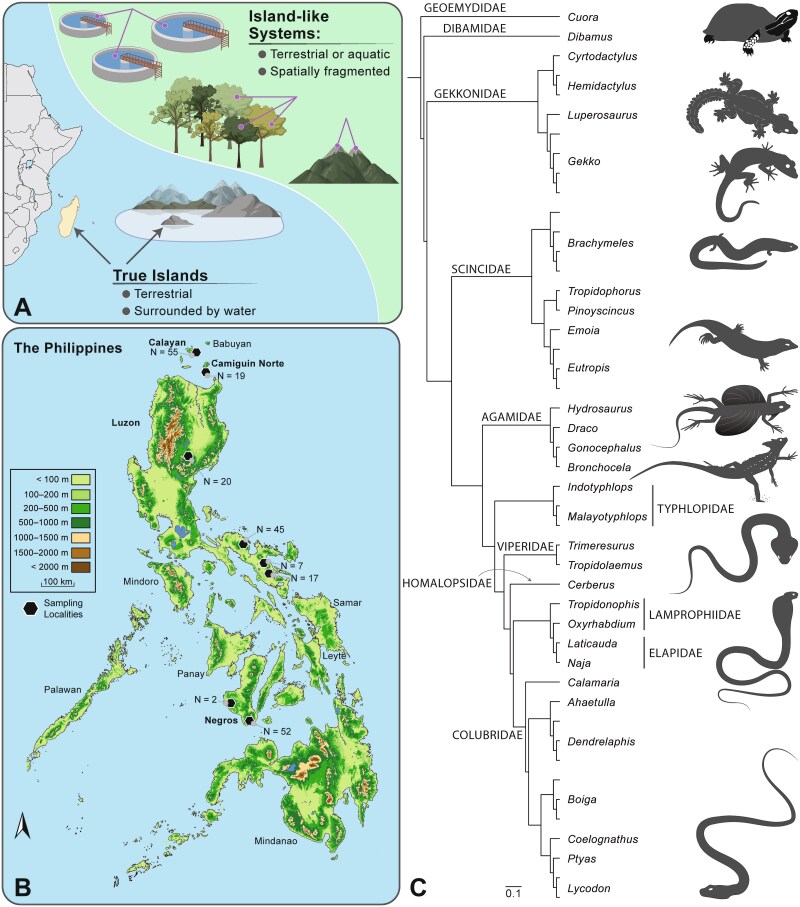
(A) Graphical representation of the characteristics that distinguish a true island from an island-like system (ILS). Examples of ILS include wastewater tanks, trees, and high elevation “sky” islands. This figure was created with BioRender. (B) Map of the Philippines with black hexagons depicting the eight sites where reptiles were sampled. Gut microbiome sample sizes for each site are provided for reference. (C) Phylogenetic relationships between the focal host taxa depicted using a cladogram obtained through Open Tree of Life [80]. Each branch represents a unique, sampled species, internal branches labeled with family names, clades labeled by genus name. Illustrations showing examples of body form diversity across sampled genera.

Islands have long been recognized as ideal, replicative “natural laboratories” with their well-defined land-positive boundaries, diversity of ecological and geographic characteristics (e.g. land area, degree of isolation, and topographic complexity), and impressive levels of floral and faunal endemism. It is for these reasons that islands and island systems (i.e. archipelagos) have provided the template for the development and expansion of the field of biogeography [1, 2], as well as processes of adaptive radiation [3–5] and community assembly [6, 7]. Indeed, the study of island systems laid the foundation for the development of the Equilibrium Theory of Island Biogeography [1] which has served as a paradigm to describe patterns of diversity within a myriad of plant and animal taxa spanning true, oceanic islands such as *Anolis* lizards in the Caribbean [4], drosophilid flies and honeycreeper birds in the Hawaiian islands [5], finches in the Galapagos islands [3], flora in Macaronesia [8], and reptiles on the Indonesian island of Sulawesi [9]. Shortly after its original description, MacArthur and Wilson [10] noted the Equilibrium Theory of Island Biogeography could be applied to many other kinds of isolated habitats as well, not just “true” oceanic islands. These “island-like” systems (ILS) share similarities with true oceanic islands as they are spatially fragmented, separated by barriers, and limited in their connectivity [11] (Fig. 1A). However, ILS may take on a terrestrial, freshwater, marine, or biotic form, while true islands are, by definition, isolated fragments of land that are surrounded completely by water in the ocean or a body of freshwater [11] (Fig. 1A). Although the majority of island biogeography studies to date have focused on true islands and island archipelago systems [7, 11, 12], there exists a growing body of literature that has utilized ILS such as high elevation islands (i.e. “sky islands”) [13, 14], inselbergs [15], lakes [14, 16], and even hydrothermal vents [17] to explore the biogeographic and ecological processes that shape the diversity of floral and faunal communities. However, an increasing number of studies have incorporated ILS within biogeographic studies of microbial communities.

To date, several studies have employed fundamental theories in island biogeography and ecology to help explain patterns of microbial community assembly and diversity across various terrestrial (e.g. leaves, trees) and aquatic ILS (e.g. lakes, pools, hot springs, wastewater tanks) [18–23]. Results from these studies indicate that microbial diversity tends to increase with “island” size (i.e. the species–area relationship) [19, 24], and microbial community similarity and diversity often decline with increased distance from “mainland” populations [18, 22], which aligns with the expectations for floral and faunal communities under the Equilibrium Theory of Island Biogeography [1]. However, due to the general paucity of studies which investigate patterns of microbial diversity across true islands [25], the factors that shape the diversity of microbial communities spanning true island systems remain unclear, particularly among those associated with vertebrate hosts. Indeed, there are few comparative studies of host-associated microbial diversity among insular faunal communities [26, 27], underscoring the need for studies which investigate patterns of microbial diversity spanning true, oceanic islands, especially among symbiotic microbial communities that colonize vertebrate host organisms, referred to as microbiomes [28].

Increased research interest in the microbiomes associated with vertebrate host organisms have illuminated their fundamental roles in numerous processes associated with host health (e.g. digestion, nutrient acquisition, metabolism, immunity) [29–33]. This is particularly true for microbial communities that reside in the gut, as they play instrumental roles in mitigating the development and severity of disease [34], improving host metabolic activity in response to harsh environmental conditions [35], and regulating thermal homeostasis [31, 36]. Despite the recognition that gut microbiomes play a critical role in modulating host health, persistence, and adaptation, many studies of non-mammalian gut microbiomes include intraspecific comparisons only [32, 37], limiting our ability to identify environmental and host-associated processes that contribute to microbiome differences observed among distinct populations and communities of vertebrate taxa. As such, there exists a need for integrative investigations of microbiome variation among diverse host organisms, and a growing number of studies have applied fundamental theories in ecology and evolutionary biology to help illuminate the mechanisms that shape the assemblage and diversity of non-mammalian vertebrate microbiomes [38–45]. Therefore, we provide one of the first assessments of variation in gut microbiomes among vertebrate species distributed across a true island archipelago system in Southeast Asia, the Philippines.

Situated at the interface of the Asian and Australasian faunal zones, the Philippine archipelago has emerged as a natural laboratory for studying various evolutionary and ecological phenomena from adaptive radiation [46–48], diversification [46], and long-distance dispersal [49], to patterns of community assembly [46–48, 50] and continental and island biogeography [51]. Further, three decades of taxonomic revisions, novel species discoveries, extensive and repeated biodiversity surveys, and comprehensive reviews of the resident biodiversity have contributed to our growing understanding of the boundaries, distributions, and phylogenetic relationships of many vertebrate taxa residing within the island system [47, 52, 53]. A particularly expansive body of work has focused on describing the diversity of reptiles found within the island nation [46, 52, 53].

Non-avian reptiles (amphisbaenians, crocodilians, lizards, snakes, tuataras, turtles) are an ecologically diverse group of vertebrate organisms that inhabit a wide array of habitats, occurring on all continents except Antarctica [54, 55]. The vertebrate group comprises over 12,000 species [56] which display a myriad of adaptive traits (i.e. dietary habits, parental care, behavioral thermoregulation) [57], reproductive modes, and morphological adaptations [55]. Despite a rich history of foundational research on the group, and their continual incorporation into seminal studies in ecology and evolutionary biology (e.g. venom evolution, adaptive radiation, development, dispersal, diversification) [51, 58–63], the reptile gut microbiome literature comprises few comparative studies between diverse host organisms, and a paucity of work has investigated the processes that shape the diversity and assemblage of reptilian gut microbiomes [64, 65], especially among reptilian host species that inhabit true oceanic island systems [26]. Further, although it has been recognized that host evolutionary history influences reptile gut microbiome diversity [42, 64–66], few published studies have explored signatures of phylogenetic signal between reptilian hosts and their gut microbiomes. While more narrow taxonomic foci may yield high-resolution insight into specific host-microbe systems, broad-scale comparative analyses are indispensable for understanding the evolutionary scope and generality of host–microbiome interactions.

Here, we compare the gut microbiomes of reptilian hosts spanning four islands in the Philippines to explore patterns of insular host-associated microbiome diversity. Our findings indicate that host habitat and microhabitat explain most of the variation in gut microbiome diversity, and island size may also contribute to gut microbial community differences among our focal hosts. We also provide an assessment of phylogenetic signal between reptilian hosts and their associated gut microbial communities, sampling reptiles from two orders, 11 families, and 50 species to further examine if host evolutionary history contributes to variation in reptile gut microbiome diversity. Results from our comparative study contribute to our growing understanding of the environmental, host-associated, and biogeographic processes that shape vertebrate gut microbiome diversity and assemblage among host communities inhabiting true island systems.

## Materials and methods

### Sample collection

Fieldwork for this study was conducted during the months of March, May, and/or June from 2016–2018 on four islands in the Philippines—Calayan, Camiguin Norte, Luzon, and Negros (Fig. 1B; Supplementary Table 1). These islands are located within three distinct faunal regions [46]: Babuyan Island Group (Calayan and Camiguin Norte), Luzon faunal region (Luzon), and Negros-Panay faunal region (Negros). In total, we sampled reptile communities from nine sites distributed across the four islands (Fig. 1B): five sites on Luzon Island (North: Gattaran and Quezon; South: Albay, Labo, and Tabaco), two sites on Negros Island (Hinoba-an and Valencia), one site on Calayan Island, and one on Camiguin Norte Island. All samples collected from the Gattaran site were removed from the dataset due to poor quality sequencing data, resulting in eight focal sites being included in downstream analyses (Fig. 1B; Supplementary Table 1). To explore the geographic location hypothesis, we conducted robust analyses of microbiome diversity across various geographic scales. This included intra-island and inter-island site comparisons, total island comparisons, and faunal region comparisons (Supplementary Table 1). We also grouped the three southern Luzon sites into their own “sub-faunal region”, referred to as the Bicol, which we compared to the northern Luzon site of Quezon (Fig. 1B; Supplementary Table 1).

We swabbed 299 individuals representing two reptilian orders, 11 families, and 50 species (Fig. 1C). A complete species list is provided in Supplementary Table 1. Reptiles were categorized into four broad habitat groups, aquatic (*N* = 20; included fresh- and brackish-water associated taxa and marine species), arboreal (*N* = 162), burrowing (*N* = 57), and ground-dwelling (*N* = 60), based on personal observations and literature reviews. To conduct more fine-scale evaluations of microbiome variation across host microhabitat groups, we divided the aquatic habitat group into microhabitat subgroups including taxa associated with freshwater (*N* = 10) and those associated with marine environments (*N* = 10), and the arboreal group into microhabitat subgroups for saxicolous (*N* = 41), lower-level arboreal (*N* = 39), and upper-level arboreal (*N* = 82) species (Supplementary Table 1).

Cloacal swabbing has been shown to serve as an effective proxy for sampling gut microbiome diversity in reptiles [67]. Therefore, gut microbiome samples were collected by inserting a sterile swab (Peel Pouch Dryswab Fine Tip [MWE 113], Corsham, England or 17.5 mm, Puritan Medical Products, Guilford, ME, USA) into the cloaca of the individual for approximately 3–4 seconds, twirling the swab 5–7 times. All swabs were preserved and stored in deoxyribonucleic acid (DNA)/ribonucleic acid (RNA) Shield (Zymo Research Products, Irvine, CA, USA) at the time of collection at ambient temperature in field conditions until returned to the United States (~10–21 days) where they were stored in a − 20°C freezer until DNA extraction. After the microbiome swab was collected, body mass (in grams) of the animal was measured using a digital scale and snout–vent length (in millimeters) was collected using tape measures or small rulers (~15 cm). All samples were collected in accordance with the University of Oklahoma’s Institutional Animal Care and Use Committee (IACUC Permit Nos.: R17–019). Field collection and export permits were provided by the Biodiversity Management Bureau (BMB) of the Philippine Department of Environment and Natural Resources (DENR) Nos. 260 (Renewal) and 273 (Renewal).

### Deoxyribonucleic acid extraction and sequencing

Genomic DNA was extracted from 299 swabs using Xpedition™ Soil/Fecal DNA MiniPrep kits (Zymo Research Products, Irvine, CA, USA) in the Shared Genomics Core facilities of the Sam Noble Museum. Ten negative controls (i.e. Xpedition™ Soil/Fecal DNA MiniPrep reagents only without a swab sample) were extracted alongside our 299 focal samples. We amplified and sequenced the 299 gut microbiome samples, 10 extraction negative controls, and two polymerase chain reaction (PCR)-negative controls using methods outlined in Smith *et al.* [42] and described here in brief. We used a one-step PCR method to amplify the V4 hypervariable region of the 16S ribosomal RNA (rRNA) gene using primers described in Kozich *et al.* [68]. After bead clean-up (KAPA Pure Beads; Roche Sequencing Solutions, Pleasanton, CA, USA), we quantified each sample and normalized to 10 nM of DNA before pooling the samples into a single sterile, 1.5 ml microcentrifuge tube. Sequencing was performed at the University of Oklahoma Consolidated Core Lab using the 2 × 250 bp paired-end sequencing on two runs of an Illumina MiSeq.

### Sequence analysis

Adapter sequences described in Kozich *et al.* [68] were trimmed from the paired-end raw sequencing reads using AdapterRemoval v2 and the following parameters—minquality: 30; trimqualities, maxns: 0; trimns, threads: 18 [69]. The sequences were then imported into QIIME 2 [70] where de novo chimera checking, and removal, was performed on 6,616,892 raw sequences using UCHIME [71]. This process removed around 47,000 reads, and 5,070,909 nonchimeric sequences were grouped into 50,124 operational taxonomic units (OTUs) with a closed-reference OTU database at 97% sequencing similarity using VSEARCH [72] against the Silva database (version 138) [73], indicating that nearly 80% of the raw sequences were grouped successfully into OTUs. There were 37 samples from the Quezon and Gattaran sites that were removed due to poor sequencing data (Supplementary Table 1). Once removed, 262 samples, 38,496 OTUs, and 4,369,039 sequences remained. We rarefied the remaining 262 samples to a sequencing depth of 1,000 sequences for downstream analyses which removed 45 additional samples and three host species from the dataset due to low sequence counts, leaving 217 samples and 13,288 OTUs to be used in the focal analyses (Supplementary Fig. 1). Raw sequence data generated in this study can be found in the Sequence Read Archive (SRA) under BioProject PRJNA1071189.

### Statistical analysis

The final dataset consisted of 217 post-rarefaction samples that were incorporated into downstream analyses. While the number of aquatic, ground-dwelling, and burrowing post-rarefaction samples were fairly consistent across islands: Calayan (*N* = 9, *N* = 13, and *N* = 7, respectively), Camiguin Norte (*N* = 0, *N* = 2, and *N* = 2, respectively), Luzon (*N* = 10, *N* = 13, and *N* = 16, respectively), and Negros (*N* = 1, *N* = 7, and *N* = 8, respectively). The sample sizes for arboreal reptiles varied across islands, with Luzon (*N* = 50) and Negros (*N* = 38) having higher sample sizes than Calayan (*N* = 26) and Camiguin Norte (*N* = 15). Some host species included in the broader “aquatic” habitat group are considered fully aquatic (e.g. *Cerberus rynchops* and *Laticauda laticaudata*) while others are found in or around freshwater or brackish water environments (e.g. *Cuora amboinensis*, *Emoia atrocostata*, and *Tropidonophis negrosensis*). We applied a suite of analytical techniques to determine the variables that contributed to gut microbial differences observed among our 47 reptile host species. Alpha (within sample) diversity was obtained via QIIME 2 [70] and measured using three metrics: Observed OTUs, Shannon diversity, and Faith’s phylogenetic diversity (PD). Alpha diversity was compared initially using univariate analyses and the alpha-group-significance plugin in QIIME 2, which performed pairwise Kruskal–Wallis tests to determine significant differences between groups. A *P*-value (*p*) or *q*-value (*q*) < 0.05 was considered statistically significant [74], and when multiple pairwise comparisons were conducted, we reported *q*-values instead of *P*-values to correct for multiple tests [75]. Further, we used a data mining approach via the R package *PARTY* [76, 77] to perform a Classification and Regression Tree (CART) Analysis, and determine which variable (host species, family, suborder and order, island, county, faunal region, sub-faunal region, habitat, microhabitat, and island size) accounted for most of the variance observed in the alpha diversity of our 47 focal host species.

To determine the impact that host evolutionary history had on reptilian gut microbial diversity, we used a Phylogenetic Generalized Linear Mixed Model (PGLMM) to calculate phylogenetic signal (*h^2^*) using the R packages *brms* [78] and *ape* [79]. We tested for phylogenetic signal using the three alpha diversity metrics, two beta diversity metrics (Unweighted- and Weighted-Unifrac), and a reptile host phylogeny obtained through Open Tree of Life (Fig. 1C) [80]. To compare the relative abundances of Proteobacteria and Firmicutes across host families, we generated a boxplot visualization using the *ggplot2* package in R [77, 81]. We tested for significant differences in the relative abundances of these phyla between the different host families and host suborders using an analysis of variance (ANOVA) followed by a post-hoc Tukey test in R. Further, we used the lm() function and *ggplot2* package in R to visualize the relationship between the relative abundance of Campylobacterota and two alpha diversity metrics: Shannon diversity and Faith’s PD across all samples. We also assessed the relationship between log-transformed host body mass and microbiome diversity using a PGLMM. Default PGLMM parameters were used, and our random effects were host species and host phylogeny. Further, we used an ANOVA, and a post-hoc Tukey test in R, to evaluate if log-transformed host body mass varied significantly between the different islands we sampled.

Beta diversity (among samples) analyses were conducted using the beta-group-significance plugin in QIIME 2 and a *q* < 0.05 was considered statistically significant [74]. We used two phylogenetic measures of microbial community beta diversity, Unweighted- and Weighted-Unifrac [82, 83], and group significance was tested using pairwise permutational multivariate analysis of variance (PERMANOVA) [84]. Beta diversity results were visualized via principal coordinate analysis (PCoA) using the *tidyverse* [85] and *qiime2R* [86] packages in R. We reported *q*-values instead of *P*-values to correct for multiple tests [75].

## Results

**Figure 2:**
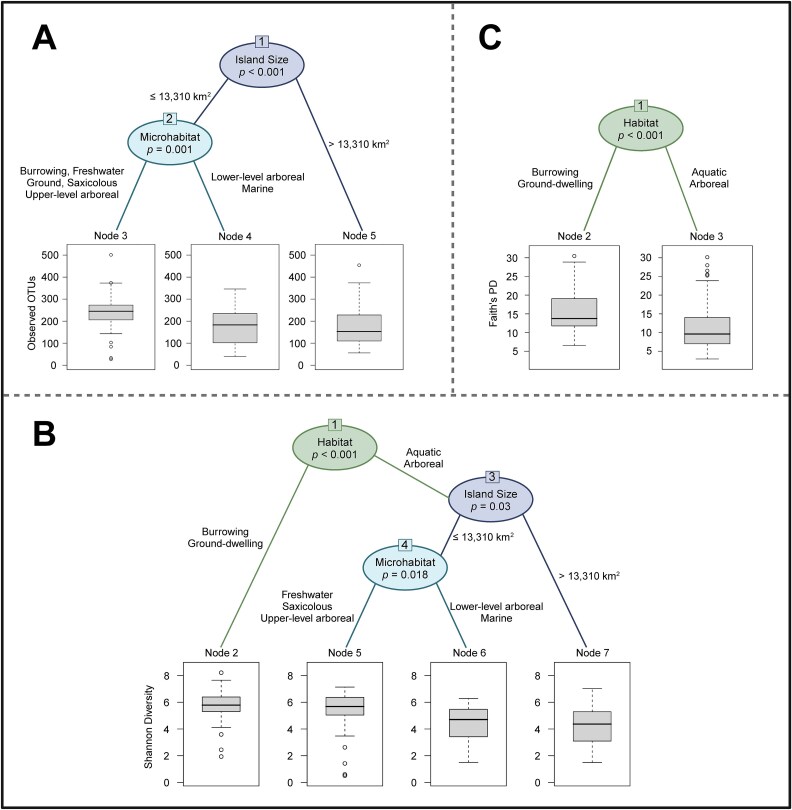
Results of the CART analysis using three alpha diversity metrics: (A) Observed OTUs, (B) Shannon Diversity, and (C) Faith’s PD. The CART analysis determined that host habitat was the primary contributor to gut microbiome differences according to the Faith’s PD and Shannon Diversity analyses. The Observed OTUs analysis indicated that island size explained most of the variation in microbiome diversity, with individuals from smaller islands (i.e. ≤13,310 km^2^) having more diverse microbiomes when compared to reptiles from the largest island of Luzon. A similar pattern was observed in the Shannon Diversity analysis, which found that island size explained some of the variance in microbiome diversity, followed by microhabitat. Microhabitat was also determined to be the secondary contributor to microbiome differences within the Observed OTUs analysis. This figure was created with BioRender.

Our focal dataset consisted of 217 gut microbiome samples representing two host orders, three suborders, 10 host families, and 47 host species spanning eight sites across four islands in the Philippines (Fig. 1; Supplementary Table 1). Results from our CART analyses revealed that host habitat, island size, and host microhabitat were key contributors to gut microbiome alpha diversity differences among our focal reptile species. For Observed OTUs, island size explained most of the variance, with microbiomes from hosts on the largest island, Luzon, being less diverse than those from hosts on the smaller islands of Calayan, Camiguin Norte, and Negros (≤13,310 km^2^; Fig. 2A; Supplementary Table 2). Host microhabitat explained the remaining variance, with the microbiomes of burrowing, freshwater, ground, saxicolous, and upper-level arboreal reptiles being significantly more diverse than the microbiomes of lower-level arboreal and marine species (χ^2^ = 65.863, *P* = 0.001; Fig. 2A). These differences were further confirmed by the results of our beta diversity analyses (Supplementary Table 3) and univariate Kruskal–Wallis tests (Supplementary Fig. 2A).

When analyzing Shannon Diversity, host habitat explained most of the variance, with microbiomes of burrowing and ground-dwelling hosts being more diverse than those of aquatic and arboreal species (χ^2^ = 73.874, *P* < 0.001; Fig. 2B). Island size also contributed to Shannon Diversity differences observed among our focal samples (χ^2^ = 40.381, *P* = 0.03; Fig. 2B). Similar to results from the Observed OTUs CART analysis, microbiomes from hosts on smaller islands (≤13,310 km^2^) were more diverse compared to those of hosts from the large island of Luzon (Fig. 2B). Host microhabitat explained the remaining variance in Shannon Diversity, with freshwater, saxicolous, and upper-level arboreal hosts having more diverse microbiomes than those of lower-level arboreal and marine reptiles (χ^2^ = 40.135, *P* = 0.018; Fig. 2B). Results from our univariate Kruskal–Wallis analysis supported these findings, as both lower-level arboreal and marine hosts harbored significantly less diverse gut microbiomes when compared to the microbiomes of burrowing and ground-dwelling hosts (Supplementary Fig. 2B). Further, the gut microbiomes of marine reptiles were significantly less diverse than the microbiomes of saxicolous and upper-level arboreal hosts (Supplementary Fig. 2B). Our univariate Kruskal–Wallis comparisons of microbiome diversity across host habitat groups also indicated that the microbiomes of aquatic individuals had significantly lower Shannon Diversity when compared to burrowing and ground-dwelling reptilian microbiomes (H = 13.969, *q* = 0.0006 and H = 15.476, *q* = 0.0005, respectively). Similarly, microbiomes of arboreal hosts had significantly lower Shannon Diversity when compared to those of burrowing and ground-dwelling hosts (Supplementary Fig. 3A). Results from our beta diversity analyses further confirmed these observed differences in microbiome diversity between distinct host habitat and microhabitat groups (Supplementary Table 3).

For Faith’s Phylogenetic Diversity (PD), host habitat explained all of the gut microbiome variance, with burrowing and ground-dwelling reptiles having more diverse microbiomes than aquatic and arboreal species, which was further confirmed by our univariate Kruskal–Wallis test (Fig. 2C; Supplementary Fig. 3B) and beta diversity analysis (Supplementary Table 3). Results from our Kruskal–Wallis test also indicated that aquatic reptiles harbored significantly less-diverse microbiomes than burrowing species (H = 8.879, *q* = 0.006). Further, arboreal individuals had significantly lower microbial diversity when compared to burrowing and ground-dwelling reptilian microbiomes (H = 18.222, *q* = 0.0001 and H = 10.282, *q* = 0.004, respectively; Supplementary Fig. 3B).

We observed that some gut microbiome samples from arboreal and aquatic hosts were over dominated by Campylobacterota (50–96%, *N* = 9); therefore, we ran a simple linear regression to test if the relative abundance of Campylobacterota correlated with both Shannon Diversity and Faith’s PD. Our results indicated that samples with higher relative abundances of Campylobacterota had lower Shannon Diversity (R^2^ = 0.243, *P* = 1.03 × 10^−14^; Supplementary Fig. 4A). Similarly, when we visualized the relationship between the relative abundance of Campylobacterota and Faith’s PD, we found a slight negative relationship, indicating that an overabundance of Campylobacterota may have contributed to overall lower gut microbiome alpha diversity within our dataset (R^2^ = 0.044, *P* = 0.002; Supplementary Fig. 4B).

Previous studies have found a positive relationship between host body mass and microbial diversity [40, 87]. Additionally, island size has been linked to insular reptilian body size variation, with more pronounced differences observed among reptiles inhabiting smaller islands [88]. Therefore, to rule out this potential confounding variable, we assessed the relationship between log-transformed host body mass and microbiome diversity using a PGLMM (Supplementary Table 1). Our results indicated that there was a negligible relationship between log-transformed host body mass and two of the diversity metrics: Observed OTUs: β = 0.01, 95% CI [−7.44, 8.13] and Shannon Diversity: β = −0.03, 95% CI [−0.23, 0.15]. However, we found a slight positive relationship when we compared log-transformed host body mass and Faith’s PD (β = 0.11, 95% CI [−0.68, 0.85]). Further, log-transformed host body mass was not significantly different between the four islands sampled (Supplementary Results).

Our CART analyses indicated that host order, host suborder, host family, and host species did not contribute significantly to microbiome alpha diversity variation (Fig. 2). However, we found that microbiome alpha diversity was significantly higher among members of the suborder Lacertilia (lizards) when compared to Serpentes (snakes) across all measures of alpha diversity (Supplementary Table 4). Additionally, we found that the relative abundance of Proteobacteria was significantly higher in the gut microbiomes of Serpentes when compared to Lacertilia microbiomes ($\overline{\textrm{X}} $1 - $\overline{\textrm{X}} $2 = 0.220 [0.124, 0.316], *q* = 5.22 x 10^−7^). The significant differences in the microbiomes of the Serpentes and Lacertilia suborders were further supported by the results of some host family-level comparisons of alpha diversity. For example, gut microbiome Observed OTUs and Shannon Diversity were significantly higher among hosts from the lizard family Scincidae when compared to the microbiomes of individuals from the snake families Elapidae and Colubridae (Supplementary Table 4).

We conducted more fine-scale comparisons of gut microbiome compositions between each host family by comparing the relative abundances of two microbial phyla, Proteobacteria and Firmicutes, across host families using an ANOVA, followed by a Tukey post-hoc test. Our results indicated several significant differences (Supplementary Fig. 5; Supplementary Table 5). Indeed, the relative abundance of Proteobacteria within samples from the lizard family Dibamidae was significantly lower than samples from the snake families Colubridae and Viperidae (Supplementary Fig. 5A; Supplementary Table 5). In contrast, dibamid samples had significantly higher relative abundances of Firmicutes when compared to the snake families Colubridae, Elapidae, and Viperidae (Supplementary Fig. 5B; Supplementary Table 5). Detailed descriptions of the gut microbiome compositions for each host family are included in the Supplementary Results. To explicitly test the host evolutionary history hypothesis, we used PGLMMs to quantify phylogenetic signal, and found no evidence of phylogenetic signal across all measures of alpha (Observed OTUs: *h*^2^ = 0.14, Estimated Error = 0.11; Shannon Diversity: *h*^2^ = 0.16, Estimated Error = 0.10; Faith’s PD: *h*^2^ = 0.14, Estimated Error = 0.1) or beta diversity (Unweighted-Unifrac: *h*^2^ = 0.12, Estimated Error = 0.13; Weighted-Unifrac: *h*^2^ = 0.1, Estimated Error = 0.11). Based on these findings, we rejected the hypothesis that host evolutionary history contributed significantly to gut microbiome variation among our focal samples.

To evaluate the geographic location hypothesis, we conducted extensive intra- and inter-island comparisons of gut microbiome alpha diversity across several geographic scales and found no significant differences in the gut microbiomes of individuals from the same island (i.e. intra-island; Supplementary Table 2). For example, microbiome diversity did not differ between reptiles collected from the two sites on Negros Island (Hinoba-an and Valencia) or the four Luzon Island sites (Albay, Labo, Quezon, and Tabaco; Supplementary Table 2). Further, when comparing alpha diversity of microbiome samples from hosts spanning different sites across all four islands (i.e. inter-island site comparisons), only the results of the Observed OTUs analysis indicated any significant differences (Supplementary Table 2). When we combined the site data for each island, the total inter-island comparisons of Observed OTUs indicated that individuals from Calayan, Camiguin Norte, and Negros islands all had significantly higher gut microbiome diversity when compared to reptiles from Luzon Island (H = 13.429–15.958, *q* = 0.0003–0.0005; Supplementary Table 2). Similarly, our inter-island Shannon Diversity analysis identified significant differences between the gut microbiomes of individuals from Luzon Island and both Calayan and Negros islands (Supplementary Table 2), with reptiles from Calayan and Negros islands having significantly higher gut microbiome diversity when compared to reptiles from Luzon Island. These differences were supported by our microbiome composition results which indicated that several microbial phyla, such as Desulfobacterota and Verrucomicrobiota, were found among samples from Calayan, Camiguin Norte, and Negros islands only, and were not identified within Luzon Island samples (Supplementary Results; Supplementary Fig. 6). However, analysis of Faith’s PD indicated no significant differences in gut microbiome diversity among reptilian hosts sampled across the four islands (Supplementary Table 2).

**Figure 3:**
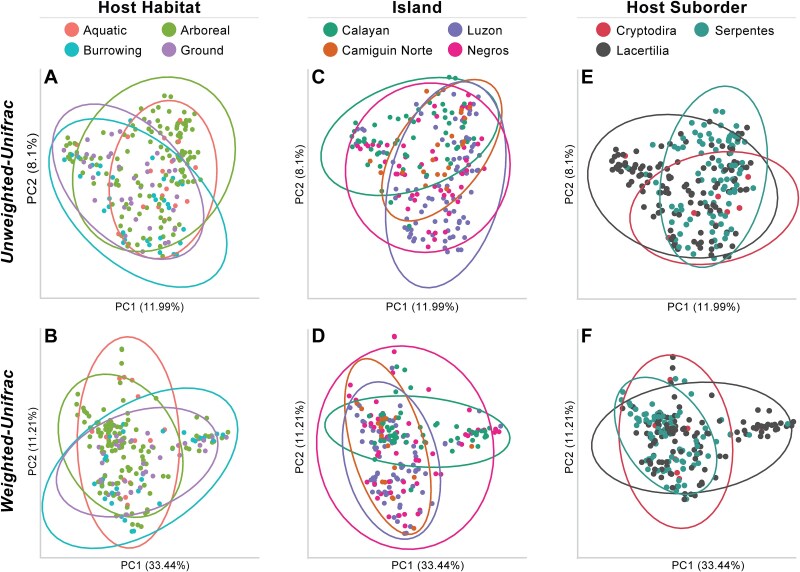
PCoA of Unweighted-Unifrac (top row) and Weighted-Unifrac (bottom row) distances for three beta diversity group comparisons: host habitat (A and B), island (C and D), and host suborder (E and F).

Results from our Unweighted-Unifrac PERMANOVA indicated that reptilian gut microbiome diversity differed significantly between all host habitat groups (pseudo-F = 1.609–3.205; *q* = 0.002–0.019; Fig. 3A). Similarly, nearly all Weighted-Unifrac PERMANOVA habitat group comparisons were significantly different (pseudo-F = 3.039–4.402; *q* = 0.006–0.028; Fig. 3B), except for burrowing vs. ground-dwelling reptiles (pseudo-F = 0.881, *q* = 0.388; Supplementary Table 3). Further, our Unweighted-Unifrac results suggested that reptilian gut microbiome diversity differed across all islands sampled (pseudo-F = 2.161–7.901; *q* = 0.001–0.002; Fig. 3C; Supplementary Table 3), and three of the four Weighted-Unifrac island comparisons indicated significant differences (Fig. 3D; Supplementary Table 3). We also found that Unweighted-Unifrac diversity differed significantly between reptiles comprising different host microhabitat groups (Supplementary Table 3). For example, burrowing reptiles had significantly different gut microbiome diversity when compared to ground, lower arboreal, upper arboreal, and marine reptiles (pseudo-F = 1.609–3.267, *q* = 0.002–0.039; Supplementary Table 3). Further, the gut microbiome diversity of marine reptiles differed significantly from the gut microbiomes of reptiles from every other microhabitat group, and these results were further confirmed by many of our Weighted-Unifrac comparisons (Supplementary Table 3). Lastly, beta diversity (both Unweighted- and Weighted-Unifrac) differed significantly between the lizard (Lacertilia) and snake (Serpentes) suborders (Fig. 3E, F; Supplementary Table 3), which was further supported by the results of several host family comparisons of beta diversity (Supplementary Table 3; Supplementary Fig. 7). For example, gut microbiome beta diversity of lizards from the families Agamidae, Gekkonidae, and Scincidae all differed significantly from gut microbiome samples collected from members of the snake families Colubridae and Elapidae (Supplementary Table 3). Further, Gekkonidae and Scincidae microbiomes also differed significantly from the gut microbiomes of viperid snakes (Supplementary Table 3).

## Discussion

Our study sampled the gut microbiomes of reptile communities spanning true oceanic islands to provide one of the first comparisons of microbiome diversity among vertebrate hosts spanning the Philippine archipelago system. We found that host habitat was a primary contributor to gut microbiome diversity differences observed among the 47 focal reptilian species, and host microhabitat also explained some of the variance (Fig. 2; Supplementary Figs. 2, 3). The CART, Kruskal–Wallis, and beta diversity analyses all confirmed that burrowing and ground-dwelling reptiles possessed significantly more diverse microbiomes when compared to aquatic and arboreal species (Fig. 2; Supplementary Fig. 3; Supplementary Table 3). Microbiome composition data further supported these findings, as some aquatic and arboreal individuals had gut microbiomes that were over dominated by Campylobacterota, and our results indicated that an overabundance of Campylobacterota had a slight negative correlation with microbiome alpha diversity. Previous findings have indicated that differences in reptile gut microbiome diversity correlated with distinct host ecologies [42, 65], and processes such as differential selection—hosts occupying similar ecological niches will be exposed to similar resident pools of microorganisms—may contribute to the observed relationship between host habitat and microbiome variation [89, 90]. Differences in host diet and resource availability may have also contributed to the significant differences we observed in the gut microbiomes of hosts occupying distinct habitats. For example, the burrowing hosts sampled in our study feed on insects, earthworms, and/or ants [91–93], whereas the marine reptiles that we sampled eat fish and other aquatic organisms (e.g. eels, crustaceans) [94]. It is well understood that host diet shapes the gut microbiome composition of reptiles [64] and future studies are needed to better understand the impact that host diet and resource availability have on the gut microbiome diversity of insular reptilian communities.

Other evolutionary mechanisms that are proposed to contribute to host–associated microbiome variation include dispersal (i.e. horizontal and vertical transmission), ecological drift, and host diversification, otherwise known as co-speciation [90]. The process of co-speciation refers to when hosts speciate over evolutionary time and their communities of symbiotic microbes also evolve in a way that matches the host phylogeny [95, 96]. This concept is also referred to as phylosymbiosis [89, 96], and many organisms have been shown to exhibit phylosymbiosis with their associated microbiomes, including insects [38, 95, 97], mammals [98, 99], sponges [100], corals [101], and plants [102]. To our knowledge, our study is the first to test this phenomenon on reptilian microbiomes. Although we did not detect signatures of phylosymbiosis among our focal samples, we did find significant differences in gut microbiome alpha and beta diversity between members of several distinct host families and two suborders. Further, results from our comparisons of Proteobacteria and Firmicutes relative abundances across host families indicated significant differences in the relative abundances of these phyla across several host families. Additionally, we found that members of the Lacertilia suborder had significantly lower relative abundances of Proteobacteria, and significantly higher overall gut microbiome diversity, when compared to Serpentes gut microbiomes. We also acknowledge that some host families had smaller sample sizes (e.g. Dibamidae, Homalopsidae, and Typhlopidae; Supplementary Table 5) than others, and the sample sizes for host suborders Lacertilia (*N* = 122) and Serpentes (*N* = 87) were higher than Cryptodira (*N* = 8). These differences in sample sizes may have impeded our ability to detect some significant differences in gut microbiome diversity between members of these host taxonomic groups. Therefore, future studies should continue sampling the microbiomes of diverse reptilian hosts and consider comparing individual microbial lineages across host taxa to conduct more fine-scale evaluations of phylosymbiosis among reptilian hosts and their associated microbiota. We argue that the rich, and often endemic, reptilian diversity of the Philippine archipelago represents an ideal system to further explore these paradigms.

Although the Philippines has long been considered an exceptional island system for investigating a myriad of ecological and evolutionary concepts [46–49, 51], few published studies to date have explored the gut microbiomes of the archipelago’s vertebrate taxa [42, 44, 103], and our study leverages fundamental theories in evolutionary biology and ecology to help identify the factors that contribute to microbiome variation among host communities spanning numerous islands in this diverse archipelago. The Philippines represents an excellent island system to conduct biogeographic studies of host–associated microbial communities in a systematic way, allowing future research to analyze community-level microbiome differences across various geographic scales. For example, there are seven defined faunal regions within the Philippines, each comprising different islands varying in size from large islands such as Luzon and Mindanao to the small islands of the Sulu faunal region [46]. Sampling host communities from a single faunal region would help control for variation in the geological history of the islands and the biogeographical history of the insular hosts, allowing for more controlled investigations of island biogeography theories within a host-associated microbiome context. Additionally, a recent review by dela Cruz *et al.* [104] highlighted the diversity of novel environmental microorganisms described within the Philippines and identified islands within the archipelago where studies of environmental microbes are limited (i.e. Babuyan Islands, Negros, Mindanao). As such, continued effort to sample environmental microbiomes across diverse islands in the Philippines will allow for future investigations to better understand the impact that environmental microbiomes may have on shaping the assemblage of vertebrate gut microbiomes. While our study utilized amplicon sequencing technologies, future studies of insular microbial communities may also consider leveraging metagenomic sequencing methodologies to gain broad insights on the potential metabolic and functional activities of these microbiomes.

Results from our study suggest that multiple factors shape the insular reptile gut microbiome; however, host habitat and host microhabitat were the primary contributors to gut microbial community differences observed among our focal samples. Comparative studies of gut microbiomes spanning complex landscapes, such as oceanic islands, allow us to apply well-established and foundational theories in ecology, evolutionary biology, and biogeography to explain patterns of host–associated microbial diversity. The investigation of reptile gut microbiomes remains critical, especially in the Philippines, where the vertebrate group continues to face challenges imposed by habitat loss, climatic changes, and the international wildlife trade.

## Supplementary Material

2025_05_05_Supp_Fig_1_Rarefaction_Curves_ycaf141

2025_08_06_SuppFig2_AlphaDiversity_ycaf141

2025_07_08_SuppFig3_Ecology_AlphaDiversity_ycaf141

2025_07_18_Supp_Fig_4_Camp_Shannon_Regression_Plot_ycaf141

2025_08_08_Supp_Fig5_HostFamily_Boxplot_ycaf141

2025_05_01_Supp_Fig6_TaxaBarplot_ycaf141

2025_05_05_Supp_Fig7_BetaDiversity_Family_ycaf141

2025_08_06_Smith_et_al_Supp_Table1_ycaf141

2025_08_06_Smith_et_al_Supp_Table2_ycaf141

2025_08_06_Smith_et_al_Supp_Table3_ycaf141

2025_08_06_Smith_et_al_Supp_Table4_ycaf141

2025_08_06_Smith_et_al_Supp_Table5_ycaf141

2025_08_06_Smith_et_al_Supp_Table5_ycaf141

2025_08_28_Smith_et_al_Clean_Supplementary_Mat_ycaf141

## Data Availability

The datasets presented in this study can be found online in the NCBI SRA under accession number PRJNA1071189.
