# Supplementary figures and images for: Host habitat shapes the gut microbiomes of insular reptilian hosts in the Philippines
Source: ISME Commun. 2025 Sep 4;5(1):ycaf141. doi: 10.1093/ismeco/ycaf141 (PMC12456179; doi:10.1093/ismeco/ycaf141)

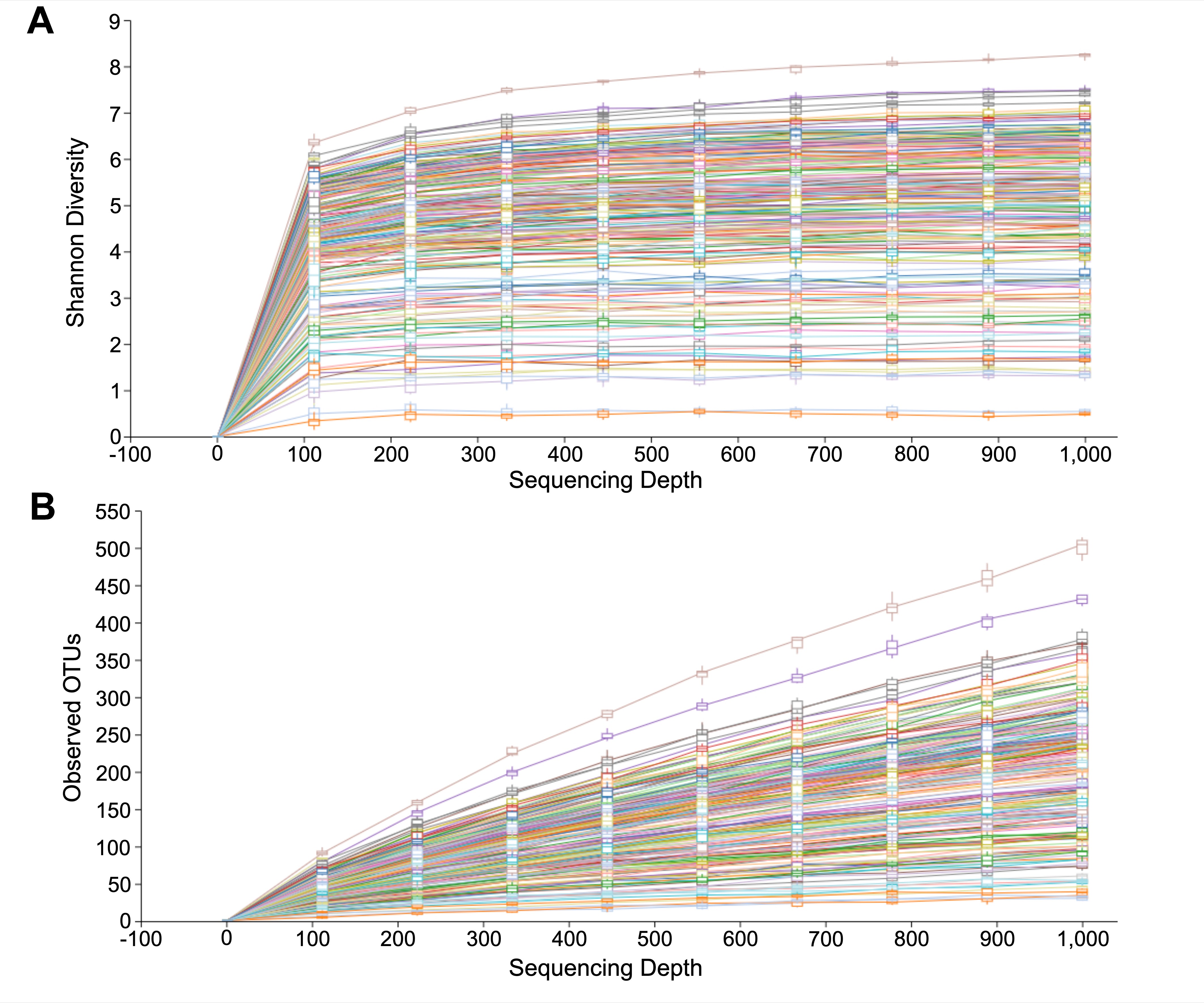

Supplement: 2025_05_05_Supp_Fig_1_Rarefaction_Curves_ycaf141 [file 2025_05_05_supp_fig_1_rarefaction_curves_ycaf141.jpeg]

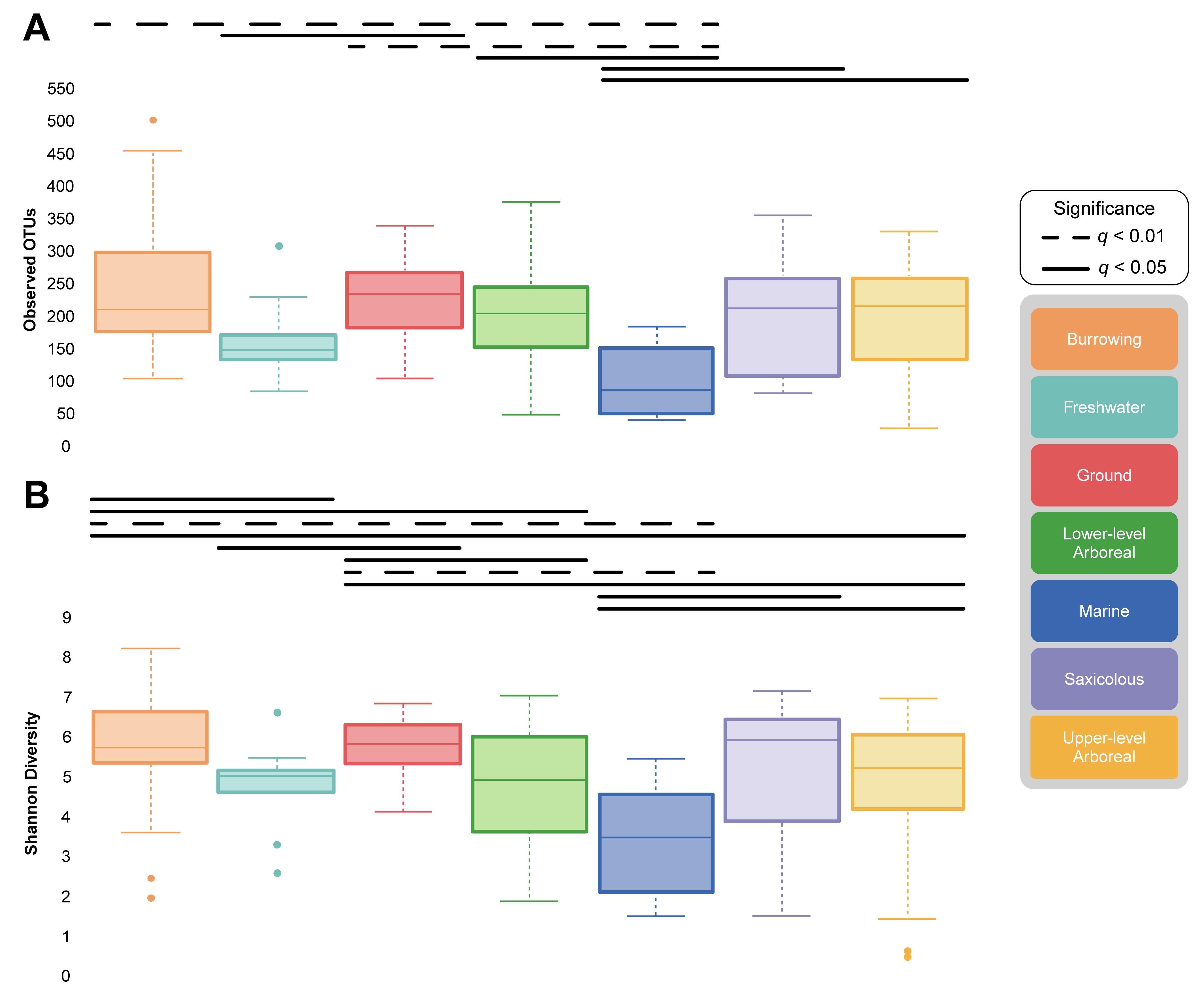

Supplement: 2025_08_06_SuppFig2_AlphaDiversity_ycaf141 [file 2025_08_06_suppfig2_alphadiversity_ycaf141.jpeg]

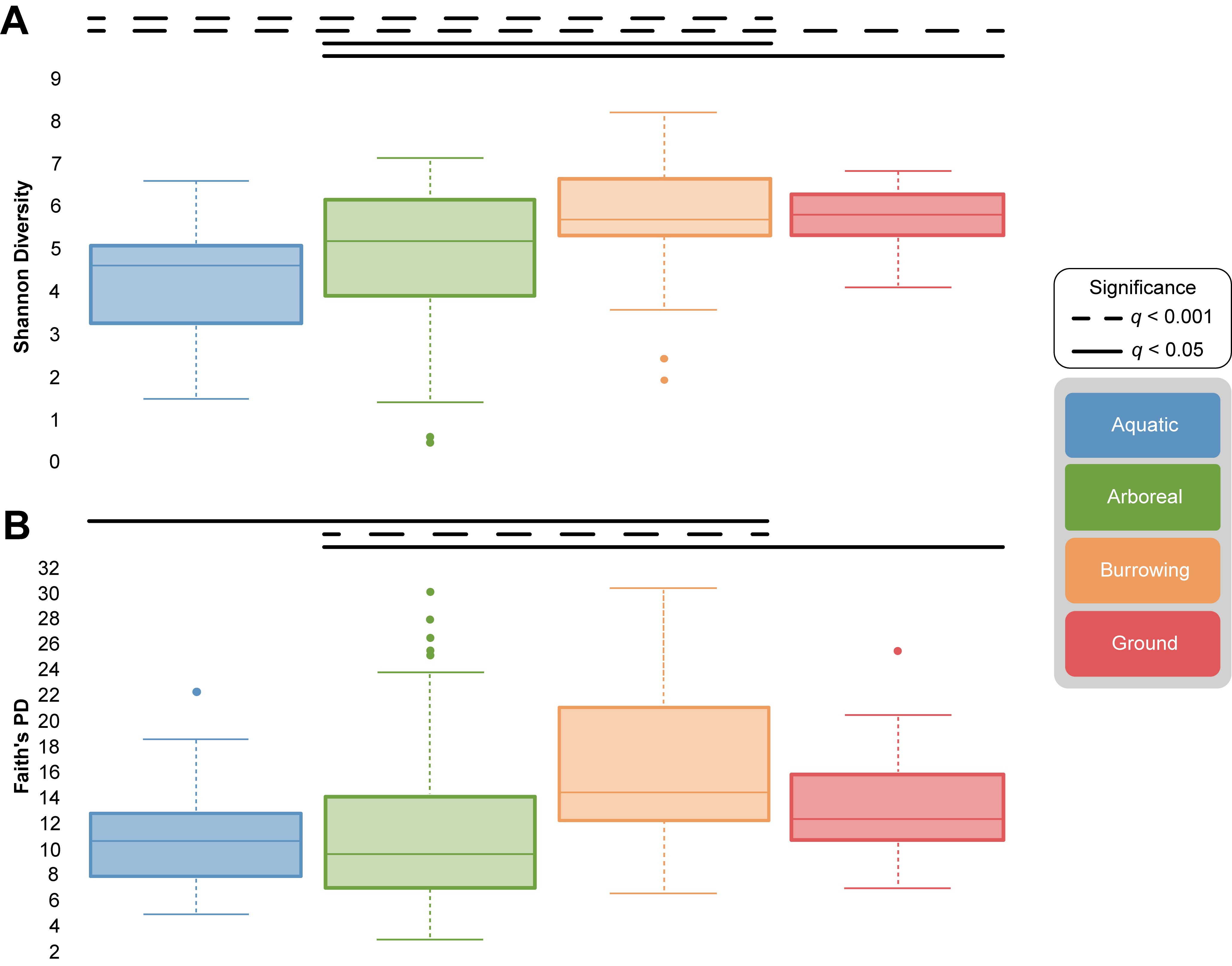

Supplement: 2025_07_08_SuppFig3_Ecology_AlphaDiversity_ycaf141 [file 2025_07_08_suppfig3_ecology_alphadiversity_ycaf141.jpeg]

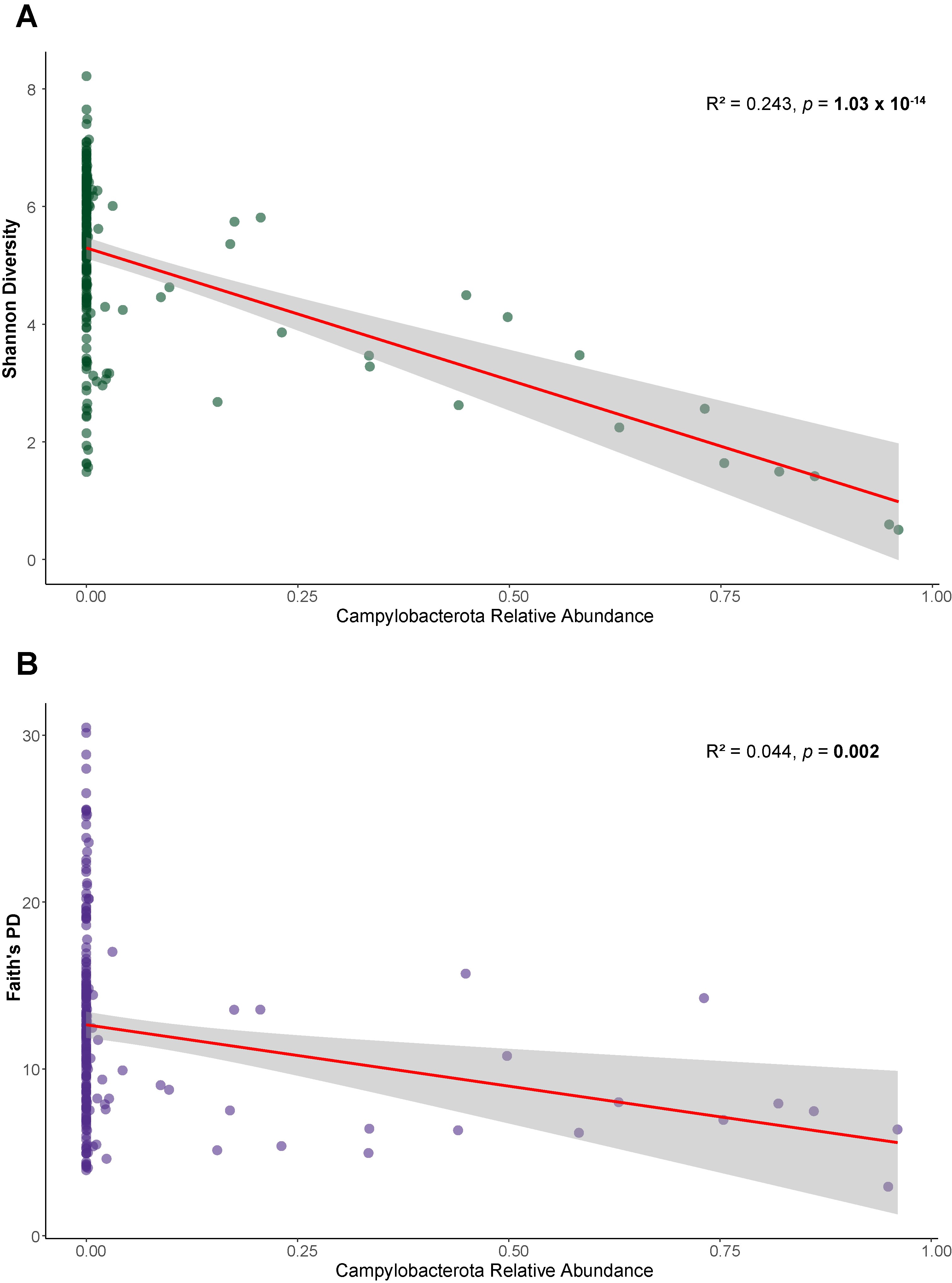

Supplement: 2025_07_18_Supp_Fig_4_Camp_Shannon_Regression_Plot_ycaf141 [file 2025_07_18_supp_fig_4_camp_shannon_regression_plot_ycaf141.jpeg]

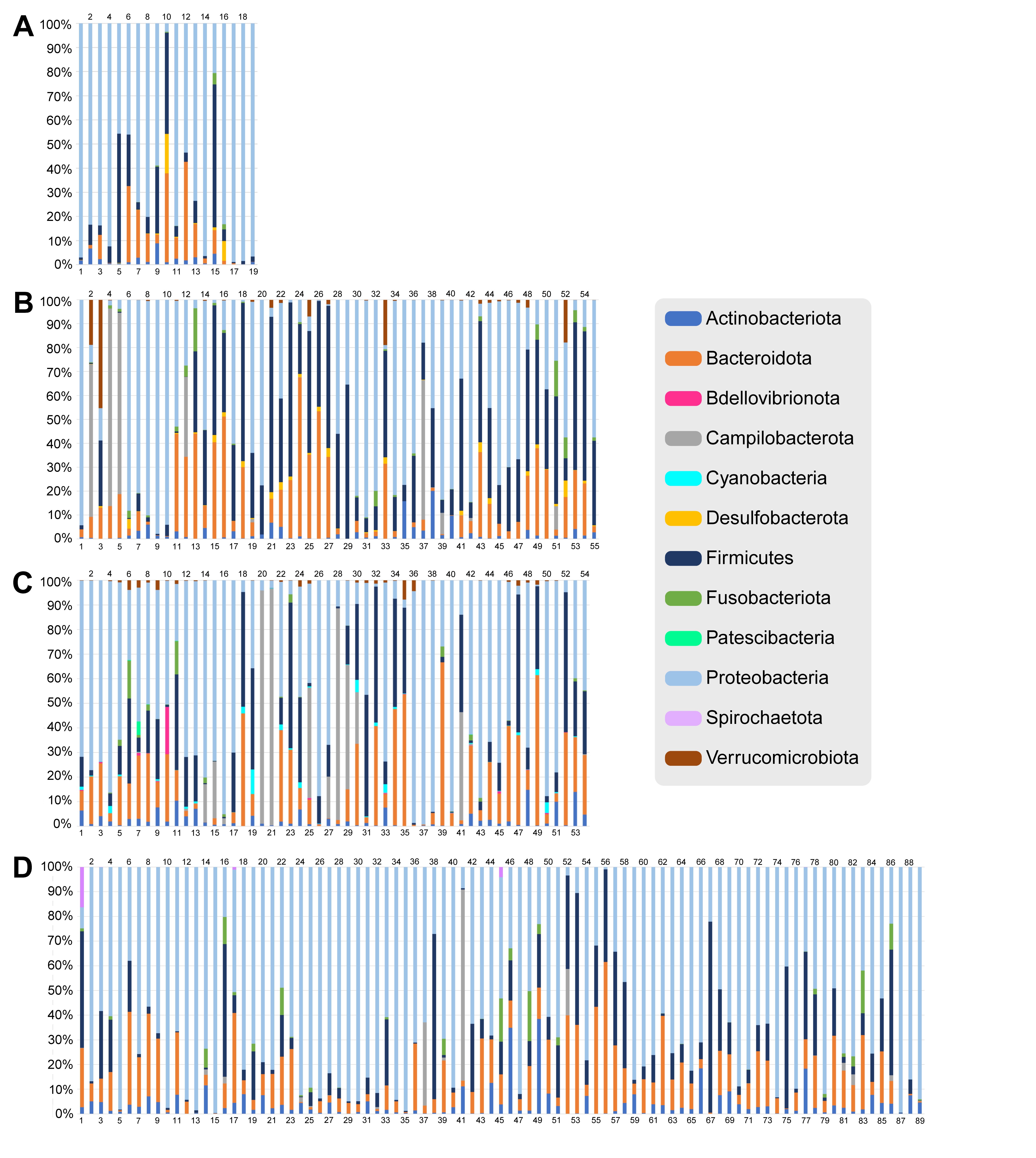

Supplement: 2025_05_01_Supp_Fig6_TaxaBarplot_ycaf141 [file 2025_05_01_supp_fig6_taxabarplot_ycaf141.jpeg]

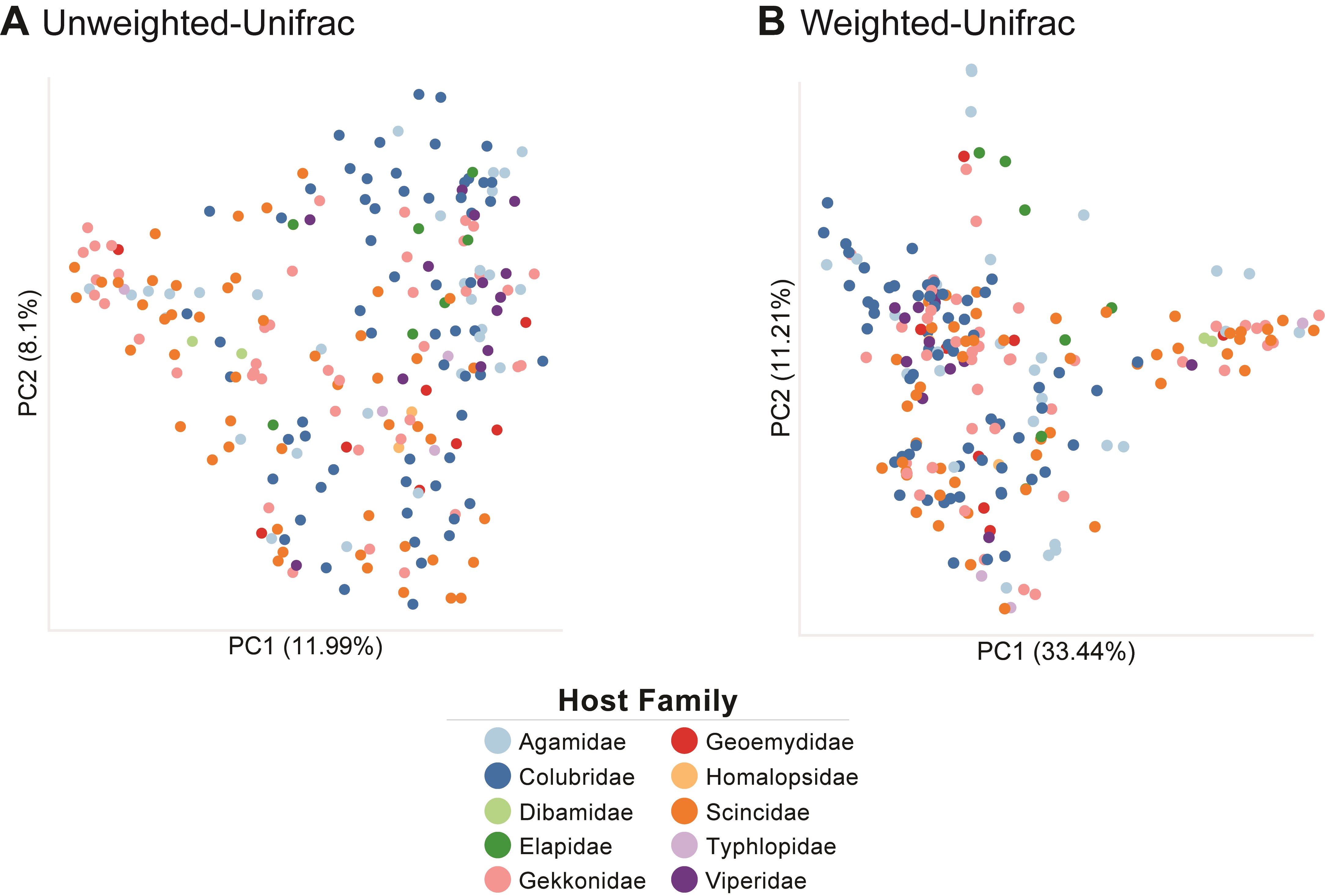

Supplement: 2025_05_05_Supp_Fig7_BetaDiversity_Family_ycaf141 [file 2025_05_05_supp_fig7_betadiversity_family_ycaf141.jpeg]
