# Supplementary material for: Host habitat shapes the gut microbiomes of insular reptilian hosts in the Philippines
Source: ISME Commun. 2025 Sep 4;5(1):ycaf141. doi: 10.1093/ismeco/ycaf141 (PMC12456179; doi:10.1093/ismeco/ycaf141)
Supplement: 2025_08_28_Smith_et_al_Clean_Supplementary_Mat_ycaf141 [file 2025_08_28_smith_et_al_clean_supplementary_mat_ycaf141.pdf]

Supplementary Materials

Host habitat shapes the gut microbiomes of insular reptilian hosts in the Philippines

Sierra N. Smith [1,2,3*], Jason B. Fernandez[4], Cameron D. Siler [1,2]

[1]Sam Noble Oklahoma Museum of Natural History, 2401 Chautauqua Ave., Norman, OK 73072, USA

[2]School of Biological Sciences, University of Oklahoma, 730 Van Vleet Oval, Room 314, Norman, Oklahoma 73019, USA

[3]Current: Department of Biology, University of Texas at Arlington, 501 S. Nedderman Dr. Box 19498, Life Science Bldg. Rm. 337, Arlington, Texas 76019-0498, USA

[4]Zone 1, Bogñabong, Tabaco City, Albay, Philippines

*** Correspondence:** Sierra N. Smith, Department of Biology, University of Texas at Arlington, 501 S. Nedderman Dr. Box 19498, Life Science Bldg. Rm. 337, Arlington, Texas 76019-0498, USA, sierrasmith220@gmail.com

SUPPLEMENTARY RESULTS

Detailed microbiome composition results

Among most of our focal gut microbiome samples (N = 212), Proteobacteria emerged as the most abundant phyla (56.83%). Three other dominant (relative abundance ≥ 1%) phyla were found among the majority of samples: Actinobacteriota (4.68%; N = 136), Bacteroidota (16.54%; N = 184), and Firmicutes (21.41%; N = 179). Viperidae gut microbiome samples were dominated by Proteobacteria

(84%), but also contained Bacteroidota (11%), Firmicutes (4%), and Actinobacteriota (2%) in lower abundances. Similarly, Colubridae samples had high relative abundances of Proteobacteria (72%), and Firmicutes (10%), Bacteroidota (9%), Actinobacteriota (4%), and Verrucomicrobiota (1%) made up smaller portions of the overall composition. Most samples from the family Homalopsidae contained only Proteobacteria (61%), Bacteroidota (19%), and Firmicutes (17%), whereas six microbial phyla, Actinobacteriota (3%), Bacteroidota (17%), Campylobacterota (29%), Firmicutes (19%), Fusobacteriota (3%), and Proteobacteria (44%) dominated the gut microbiomes of most lizards in the family Agamidae. Samples from the Dibamidae family had the lowest percentage of Proteobacteria (4%), and were instead dominated by Firmicutes (54%), Bacteroidota (36%), Desulfobacterota (8%), and also contained Verrucomicrobiota (2%) and Cyanobacteria (1%). Campylobacterota were the most abundant phylum among Elapidae samples (63%) which also contained Proteobacteria (23%), Bacteroidota (21%), Verrucomicrobiota (13%), Firmicutes (8%), Fusobacteriota (2%), and Actinobacteriota (1%). Two samples from the turtle family Geoemydidae and the lizard family Gekkonidae had high abundances of Campylobacterota (Geoemydidae: 33% and 73%; Gekkonidae: 44% and 58%), but most Geoemydidae samples were dominated by Proteobacteria (45%), Firmicutes (23%), Bacteroidota (13%), Actinobacteriota (3%), and Bdellovibrionota (2%) while Gekkonidae samples comprised Proteobacteria (49%), Firmicutes (24%), Bacteroidota (16%), Actinobacteriota (4%), Cyanobacteria (1%), and Desulfobacterota (1%). Similar to compositions of Geoemydidae and Gekkonidae samples, Typhlopidae snakes possessed low relative abundances of Bdellovibrionota (2%) and Desulfobacterota (1%), respectively, but they also contained Proteobacteria (55%), Bacteroidota (30%), Firmicutes (10%), and Actinobacteriota (3%). Although the average relative abundance was low (1%), Acidobacteriota was found among Scincidae samples only which also contained six other phyla: Proteobacteria (48%), Firmicutes (26%), Bacteroidota (17%), Actinobacteriota (3%), Verrucomicrobiota (2%), and Desulfobacterota (1%).

Microbiome composition results for the four focal islands

Desulfobacterota was detected among Calayan and Camiguin Norte samples, and Verrucomicrobiota was present within the gut microbiomes of individuals from Calayan and Negros islands. Desulfobacterota and Verrucomicrobiota have been identified as dominant members of some snake species [1–3]; therefore, it is not surprising that these phyla were detected within our focal samples. Further, Bdellovibrionota, Cyanobacteria, and Patescibacteria were found within samples from Negros Island only (Supplementary Fig. 4). Patescibacteria has been identified as a dominant member of the *Laticauda laticaudata* oral microbiome, but no other studies to date have reported Patescibacteria within reptilian gut microbiome samples, but members of this large microbial radiation are found in various aquatic environments such as groundwater [4] and seawater [5], and activated sludge collected from wastewater tanks [6]. Similarly, Bdellovibrionota has not yet been reported as a dominant member of the reptilian gut microbiome, but members of the phylum are found commonly within the environment (i.e., soil, freshwater, ocean). In contrast, other reptilian gut microbiome studies have detected Cyanobacteria in low relative abundances [1, 7, 8]. Only one phylum was unique to samples from Luzon Island (Spirochaetota; Supplementary Fig. 4), and members of this phylum have been found within the gut microbiomes of other reptile species [7–10].

Correlation of host body mass across islands

We performed an ANOVA and, subsequently, a post-hoc Tukey test on log-transformed host body mass across islands to evaluate if host body size differed significantly across islands, and we found no significant differences in log-transformed host body mass across islands (Luzon vs. Negros: adjusted p-value = 0.955; Luzon vs. Calayan: adjusted p-value = 0.999; Luzon vs. Camiguin Norte: adjusted p-value = 0.978; Calayan vs. Negros: adjusted p-value = 0.961; Camiguin Norte vs. Negros: adjusted p-value = 0.886; Calayan vs. Camiguin Norte: adjusted p-value = 0.982)

SUPPLEMENTARY FIGURE LEGENDS

Supplementary Figure 1 | Rarefaction curves based on (A) Shannon Diversity and (B) Observed OTUs alpha diversity metrics. Each curve is representative of a single swab. Based on these curves, we rarefied all samples to a sequencing depth of 1,000 sequences.

Supplementary Figure 2 | (A) Observed OTUs and (B) Shannon Diversity comparisons between different host microhabitat groups. The different groups are distinguished based on color with burrowing = orange, freshwater = teal, ground-dwelling = red, lower-level arboreal = dark green, marine = dark blue, saxicolous (rock-dwelling) = purple, and upper-level arboreal = yellow. Horizontal bars represent significant differences between microhabitat groups, and the position of the bars are indicative of the two microhabitat groups being compared. A solid bar indicates a significant difference of $q < 0.05$, while a dashed bar represents a significant difference of $q < 0.01$. (A) Burrowing and ground-dwelling reptiles had significantly more Observed OTUs within their gut microbiomes when compared to the gut microbiomes of marine species. The microbiomes of marine species were also significantly less diverse than the microbiomes of individuals from both arboreal microhabitat groups (lower and upper) and the saxicolous group. (B) Microbiome Shannon Diversity was significantly higher among burrowing reptiles when compared to the microbiomes of all other microhabitat groups except for ground-dwelling and saxicolous species. Similarly, ground-dwelling reptiles possessed significantly more diverse microbiomes than individuals from all other microhabitat groups except burrowing and saxicolous species. Microbiomes of marine species were also significantly less diverse than the microbiomes of saxicolous and upper-level arboreal individuals.

Supplementary Figure 3 | Alpha diversity comparisons between the four host habitat groups using (A) Shannon Diversity and (B) Faith's Phylogenetic Diversity (PD). Different host habitat preferences are distinguished by color with aquatic = blue, arboreal = green, burrowing = orange, and ground-dwelling = red. Horizontal bars represent significant habitat group differences, and the position of the bars are indicative of the two habitat groups being compared. A solid bar indicates a

significant difference of $q < 0.05$, while a dashed bar represents a significant difference of $q < 0.001$. (A) Shannon diversity was significantly lower when comparing the microbiomes of aquatic reptiles to all other habitat groups. Arboreal species also possessed significantly lower gut microbiome diversity when compared to burrowing and ground-dwelling reptiles, but ground-dwelling and burrowing species did not differ from one another. (B) Similar results were found using Faith's PD, with the exception of aquatic and arboreal reptiles which did not have significantly different gut microbiome diversity.

Supplementary Figure 4 | Simple linear regressions depicting the correlation between the relative abundance of Campylobacterota and two measures of microbiome alpha diversity: (A) Shannon Diversity indicated with green dots, and (B) Faith's Phylogenetic Diversity (PD) shown in purple dots across all samples.

Supplementary Figure 5 | Boxplot visualizations comparing the relative abundances of two microbial phyla: (A) Proteobacteria and (B) Firmicutes across samples representing the 10 host families which are denoted by color, and individual samples are overlayed as dots. Results of the ANOVA and subsequent post-hoc Tukey test are represented by the horizontal bars, and a solid bar indicates a significant difference of $q < 0.05$, while a dashed bar represents a significant difference of $q < 0.001$.

Supplementary Figure 6 | Relative abundances of the dominant microbial phyla present within our focal gut microbiome samples. Each vertical bar represents a single reptile individual which are assigned a number that corresponds to the sample's metadata in Supplementary Table 1. Samples are grouped based on the geographic locations where the hosts were found: (A) Camiguin Norte, (B) Calayan, (C) Negros, and (D) Luzon.

Supplementary Figure 7 | Principal coordinate analysis (PCoA) of (A) Unweighted-Unifrac and (B) Weighted-Unifrac distances across all samples, with dot color indicating the host's family.

SUPPLEMENTARY TABLE LEGENDS

Supplementary Table 1 | Metadata for 299 gut microbiome samples that were extracted, amplified, and sequenced.

Supplementary Table 2 | Alpha diversity comparisons between the sites and islands that host communities were sampled. Bold values indicate significant differences ($q < 0.05$).

Supplementary Table 3 | Results of Unweighted- and Weighted-Unifrac PERMANOVA comparisons between host microhabitats, host habitats, host families, host suborders, and islands. Bold values represent significant *q*-values ($q < 0.05$).

Supplementary Table 4 | Results of alpha diversity comparisons between host families and host suborders. Bold values indicate significant differences ($q < 0.05$).

Supplementary Table 5 | Comparisons of Proteobacteria and Firmicutes relative abundances between distinct host families using an ANOVA and subsequent Tukey's Honestly Significant Difference (HSD) test. Bold values represent significant differences ($q < 0.05$).

REFERENCES

1. Tang, W., Zhu, G., Shi, Q., Yang, S., Ma, T., Mishra, S. K., et al. (2019). Characterizing the microbiota in gastrointestinal tract segments of *Rhabdophis subminiatus*: dynamic changes and functional predictions. *MicrobiologyOpen* 8, e00789. doi: 10.1002/mbo3.789

2. Smith, S. N., Colston, T. J., and Siler, C. D. (2021). Venomous snakes reveal ecological and phylogenetic factors influencing variation in gut and oral microbiomes. *Front. Microbiol.* 12, 657754. doi: 10.3389/fmicb.2021.657754

3. Hoffbeck, C., Middleton, D. M., Nelson, N. J., and Taylor, M. W. (2023). 16S rRNA gene-based meta-analysis of the reptile gut microbiota reveals environmental effects, host influences and a limited core microbiota. *Mol. Ecol.* 32, 6044–6058. doi: 10.1111/mec.17153

4. Brown, C. T., Hug, L. A., Thomas, B. C., Sharon, I., Castelle, C. J., Singh, A., et al. (2015). Unusual biology across a group comprising more than 15% of domain Bacteria. *Nature* 523, 208–211. doi: 10.1038/nature14486

5. Takebe, H., Tominaga, K., Fujiwara, K., Yamamoto, K., and Yoshida, T. (2020). Differential responses of a coastal prokaryotic community to phytoplanktonic organic matter derived from cellular components and exudates. *Microb. Environ.* 35, ME20033. doi: 10.1264/jsme2.ME20033

6. Fujii, N., Kuroda, K., Narihiro, T., Aoi, Y., Ozaki, N., Ohashi, A., et al. (2022). Metabolic potential of the superphylum *Patescibacteria* reconstructed from activated sludge samples from a municipal wastewater treatment plant. *Microb. Environ.* 37, ME22012. doi: 10.1264/jsme2.ME22012

7. Hong, P. Y., Wheeler, E., Cann, I. K., and Mackie, R. I. (2011). Phylogenetic analysis of the fecal microbial community in herbivorous land and marine iguanas of the Galápagos Islands using 16S rRNA-based pyrosequencing. *ISME J.* 5, 1461–1470. doi: 10.1038/ismej.2011.33

8. Zhang, B., Ren, J., Yang, D., Liu, S., and Gong, X. (2019). Comparative analysis and characterization of the gut microbiota of four farmed snakes from southern China. *PeerJ* 7, e6658. doi: 10.7717/peerj.6658

9. Keenan, S. W., Engel, A. S., and Elsey, R. M. (2013). The alligator gut microbiome and implications for archosaur symbioses. *Sci. Rep.* 3, 2877. doi: 10.1038/srep02877

10. Arizza, V., Vecchioni, L., Caracappa, S., Sciurba, G., Berlinghieri, F., Gentile, A., et al. (2019). New insights into the gut microbiome in loggerhead sea turtles *Caretta caretta* stranded on the Mediterranean coast. *PLoS One* 14, e0220329. doi: 10.1371/journal.pone.0220329
